# PCR-confirmed disseminated herpes zoster initially mimicking lumbar radiculopathy in an apparently immunocompetent adult: a case report and literature review

**DOI:** 10.3389/fmed.2026.1907721

**Published:** 2026-07-30

**Authors:** Yun Gao

**Affiliations:** Lhasa People's Hospital, Lhasa, Tibet, China

**Keywords:** dermatomal pain, disseminated herpes zoster, immunocompetent, lumbar disc herniation, misdiagnosis, traction therapy

## Abstract

**Background:**

Disseminated herpes zoster (DHZ) is typically associated with impaired cell-mediated immunity and is uncommon in adults without an identified immunocompromising condition. Diagnostic delay may occur when prodromal dermatomal pain precedes rash and mimics structural spine disease.

**Case Presentation:**

A 59-year-old woman presented with 6 days of intermittent burning and needle-like pain involving the left thigh and calf and was initially diagnosed with lumbar disc herniation after lumbar CT showed multilevel degenerative disc protrusions. Lumbar traction was performed during this period, while the pain continued to worsen. Two days before admission, vesicular lesions appeared on the left thigh and rapidly extended to the back, buttock, perineum, scalp, face, trunk, and limbs. Examination showed grouped vesicles and pustules with marked tenderness but no objective focal neurological deficit. VZV DNA PCR from vesicle fluid and peripheral blood was positive. Evaluation showed no evidence of HIV infection, malignancy, diabetes, or immunosuppressive therapy. Immune testing showed no quantitative T-cell deficiency and normal immunoglobulin levels. The patient was treated with intravenous acyclovir, gabapentin, and supportive care, with cessation of new lesions after 2 days and marked clinical improvement, with no recurrence or postherpetic neuralgia at 4-week follow-up.

**Conclusion:**

This case highlights that DHZ may rarely occur in an apparently immunocompetent older adult and may initially mimic lumbar radiculopathy when imaging shows incidental degenerative changes. Acute dermatomal neuropathic pain without objective neurological deficits should prompt consideration of herpes zoster even before rash appears.

## Introduction

Reactivation of varicella-zoster virus (VZV) causes herpes zoster (HZ), which usually presents as a unilateral vesicular eruption confined to a single dermatome. Disseminated herpes zoster (DHZ), commonly defined as more than 20 vesicles outside the primary or adjacent dermatomes, is uncommon and is classically associated with impaired cell-mediated immunity, including HIV infection, malignancy, transplantation, or immunosuppressive therapy (1). In addition to its rarity in adults without an identified immunocompromising condition, DHZ may be diagnostically challenging because prodromal neuropathic pain can precede rash and mimic other disorders such as sciatica, lumbar disc herniation, or acute abdominal and neurologic conditions (2–5).

We report a case of PCR-confirmed DHZ in an apparently immunocompetent 59-year-old woman whose initial presentation mimicked lumbar radiculopathy and led to treatment for presumed lumbar disc herniation. This case is clinically instructive for three reasons: DHZ occurred without an identifiable major acquired immunocompromising condition, early symptoms were initially attributed to incidental degenerative lumbar imaging findings, and VZV DNA was confirmed in both vesicle fluid and peripheral blood. By highlighting how disseminated VZV infection may initially resemble mechanical radicular pain, this report underscores the importance of considering herpes zoster in older adults with acute unilateral dermatomal pain even before rash appears.

## Case description

A 59-year-old woman who had been experiencing left thigh pain for 6 days came to the neurology department. She had first visited an orthopedic clinic due to localized, ongoing thigh pain that was not accompanied by lower back pain, weakness, or sensory symptoms. At that time, a lumbar computed tomography (CT) scan showed central-type disc herniation at the L3/L4, L4/L5, and L5/S1 levels, along with degenerative changes in the lumbar spine. Based on these findings, the patient was treated for presumed lumbar disc herniation and underwent lumbar traction therapy; during this period, the pain continued to worsen. The pain was described as burning and needle-like paroxysms lasting about 1–2 min, recurring repeatedly, and shifting between the left thigh and calf. This brief, recurrent, and shifting pain pattern was atypical for the more commonly sustained dermatomal pain associated with herpes zoster. The pain intensity was reported as 6/10 on the visual analog scale at the initial orthopedic assessment and had increased to 9/10 by the subsequent clinical evaluation, after traction therapy had been administered. The pain was distributed mainly over the posterior left thigh with radiation to the calf. The symptoms were intermittent, were aggravated by movement and touch, and were not accompanied by definite low-back pain, sphincter dysfunction, progressive motor weakness, or objective sensory loss. Local tenderness was noted, and the straight leg raise test was recorded as positive early in the course. Although lumbar CT showed degenerative disc protrusions at L3/L4, L4/L5, and L5/S1, the clinical picture was not fully concordant with compressive radiculopathy because the pain was predominantly neuropathic in quality, objective neurological deficits were absent, and subsequent serial examinations did not demonstrate progressive motor, sensory, or sphincter impairment.

The patient observed vesicular eruptions and erythematous macules on her left thigh 2 days prior to admission to our hospital. These lesions quickly involved the back, left buttock, and perineal region (Figure 1). Other sporadic vesicles appeared on the scalp, face, trunk, and limbs within 24 h of their emergence (Figure 2), mainly on the left side, and gradually gathered in clusters. On dermatologic review, the eruption involved a presumed primary dermatomal distribution over the left lumbosacral dermatomes, with numerous lesions outside the primary and adjacent dermatomes, fulfilling the clinical definition of disseminated herpes zoster. Extra-dermatomal lesions were documented on the back, trunk, periumbilical region, lower limbs, with bilateral scattered involvement. There was no ocular or mucosal involvement, including oral or genital lesions, and no clinical evidence of visceral dissemination such as encephalitis, pneumonitis, or hepatitis. A low-grade fever (37.3 °C), headache, pruritus, and dysuria were among the systemic symptoms that the patient experienced; there was no limb weakness, sensory loss, or urine retention.

**Figure 1:**
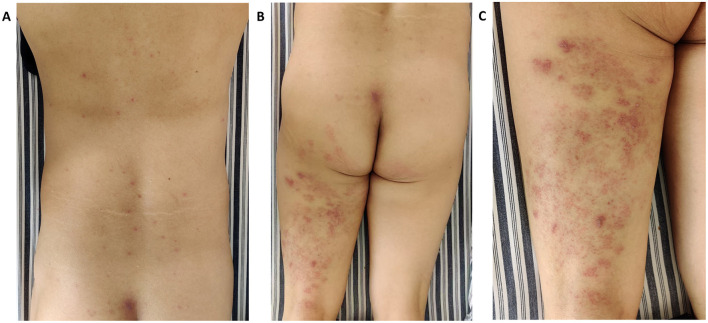
Multiple clusters of vesicles on the **(A)** back, **(B)** left buttock, and **(C)** left thigh.

**Figure 2:**
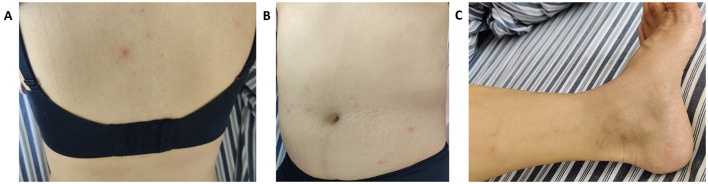
Sporadic vesicles appeared on the **(A)** back, **(B)** trunk, and **(C)** limbs.

Her past history included hypertension (in the past 10 years) and anxiety disorder; she was not receiving corticosteroids, chemotherapy, or other immunosuppressive therapy, and she had no known drug allergies.

Additional history revealed a history of uncertain prior varicella infection in childhood without herpes zoster vaccination, no recent febrile illness, trauma, or self-reported major psychological or physical stressors. She denied recent use of systemic corticosteroids, biologic agents, herbal drugs, disease-modifying immunosuppressive drugs. She was a never-smoker and reported occasional alcohol use. Her family history was noncontributory, with no known familial history of recurrent severe infections, primary immunodeficiency, hematologic malignancy, or unusually early or recurrent herpes zoster. No genetic testing was performed because the clinical history and immune evaluation did not suggest an inherited immunodeficiency.

Upon physical examination, she seemed alert, oriented, and hemodynamically stable. On initial assessment, vital signs were stable (T 36.2 °C, P 88 bpm, R 20 bpm, BP 154/89 mmHg). Examination documented patchy papules on the left flank and buttock with significant tenderness. Dermatological examination showed asymmetrically distributed purplish-red macules, papules, vesicles, and pustules on the left thigh, buttock, and perineum, with a diameter of roughly 1–10 mm. There were no indications of bleeding or necrosis, and the lesions had a characteristic erythematous halo. Additionally, there were sporadic vesiculopapular eruptions on the face, limbs, scalp, and trunk. There was no visible ulceration or secondary bacterial infection.

Focused spine and neurological examination showed an initially positive straight leg raise test that became negative on subsequent ward rounds, preserved motor strength (5/5), intact sensory testing, normal and symmetric deep tendon reflexes, a negative Babinski sign, and a normal gait. There was tenderness to light touch over the affected dermatomes. There were also no meningeal signs (Brudzinski's and Kernig's signs were negative). No bowel/bladder dysfunction was reported at presentation.

The sequence of symptoms, diagnostic evaluations, initiation of antiviral therapy, and clinical response is summarized in the timeline shown in Figure 3.

**Figure 3:**
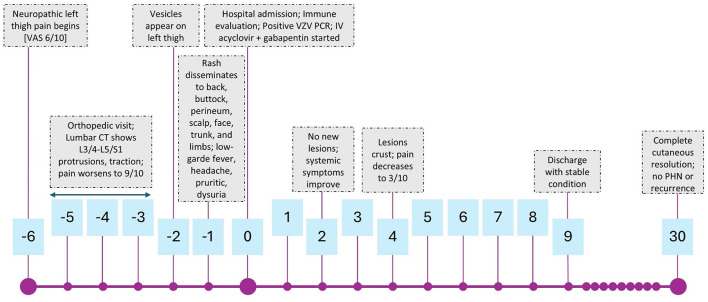
Timeline of the episode of care. PHN, Postherpetic Neuralgia; VZV, Varicella Zoster Virus; VAS, Visual Analog Score; PCR, Polymerase Chain Reaction; IV, Intravenous.

A white blood cell count of 5,200 cells/μL, comprising 66.5% neutrophils, 24.8% lymphocytes, and 6.7% monocytes, was found during the initial laboratory evaluation. Serum electrolyte levels, glucose levels, and liver and renal function tests were all within normal limits. Tumor markers such as AFP, CEA, and CA19-9 were within reference limits, and serologic testing for syphilis and HIV came back negative.

The patient had no known history of immunodeficiency, malignancy, organ transplantation, autoimmune disease requiring immunosuppression, or chronic corticosteroid exposure. Serologic testing for HIV was negative. Screening for metabolic or systemic contributors showed fasting glucose 87.4 mg/dL and HbA1c 4.7%, and there was no evidence of active malignancy on imaging. Key immune and virologic findings supporting apparent immune competence and disseminated VZV infection are summarized in Table 1.

**Table 1:** Immune and virologic workup supporting apparent immune competence and disseminated VZV infection.

Parameter	Result/interpretation
Host factors	No history of malignancy, transplantation, autoimmune disease requiring immunosuppression, or chronic corticosteroid use; no major acquired immunocompromising condition documented
HIV serology	Negative; no evidence of HIV infection
Metabolic screening	Fasting glucose 87.4 mg/dL and HbA1c 4.7%; no evidence of diabetes
Absolute lymphocyte count	2,360/μL; within reference range
CD3+ T cells	1,805.4/μL; 76.5% of total lymphocytes; within the laboratory reference range
CD4+ T cells	1,000/μL; 55.4% of CD3+ T cells; within the laboratory reference range
CD8+ T cells	725/μL; 40.17% of CD3+ T cells; within the laboratory reference range
CD4/CD8 ratio	1.38; preserved
Serum immunoglobulins	IgG 1,350 mg/dL, IgA 257 mg/dL, and IgM 153 mg/dL; within reference ranges
Flow-cytometric pattern	Mild expansion of effector-memory and activation-marker-positive T-cell subsets, compatible with recent immune activation; no evidence of quantitative major T-cell deficiency
Malignancy screen	AFP, CEA, and CA19-9 within reference limits; imaging without evidence of malignancy
VZV PCR	Positive from vesicle fluid and peripheral blood; lesion PCR provides direct etiologic confirmation and blood positivity supports viremia/systemic dissemination
HSV-1/HSV-2 PCR	Negative; alternative viral etiologies not supported

Percentages were calculated using different gating denominators: CD3+ cells are expressed as a percentage of total lymphocytes, whereas CD4+ and CD8+ cells are expressed as percentages of CD3+ T cells. Absolute counts were reported directly by the clinical laboratory.

Detailed flow-cytometric immunophenotyping showed preserved absolute CD3+, CD4+, and CD8+ T-cell counts and a normal CD4/CD8 ratio. CD3+ T cells accounted for 76.5% of total lymphocytes (1,805.4/μL); within the CD3+ T-cell gate, CD4+ and CD8+ cells accounted for 55.4% (1,000/μL) and 40.17% (725/μL), respectively. Subset analysis nevertheless showed mild expansion of CD4+ effector-memory T cells (262.15/μL, 26.21%), CD8+ effector-memory T cells (476.91/μL, 65.76%), HLA-DR+ T lymphocytes (24.18%), total CD38+ T lymphocytes (34.76%), and CD38+ cytotoxic T cells (317.07/μL, 43.72%), while naïve and central-memory compartments remained within reference ranges. This pattern is more consistent with recent antigen-driven T-cell activation during acute VZV infection, although transient functional immune dysregulation cannot be excluded.

VZV confirmation was obtained by PCR performed on vesicle fluid and peripheral blood, both of which tested positive for VZV DNA. Testing for alternative viral etiologies, including HSV-1/HSV-2, was negative. In this context, lesion-based PCR provided the most direct etiologic confirmation, whereas blood PCR positivity supported viremia in the setting of clinically evident disseminated zoster.

Based on the distribution of the rash, clinical features, and absence of any identifiable immunosuppressive condition, a diagnosis of disseminated herpes zoster in an apparently immunocompetent patient was established and confirmed by the positive VZV DNA findings. Differential diagnoses included herpes simplex virus infection, primary varicella, contact dermatitis, arthropod-bite reaction, drug-related vesiculobullous eruption, and autoimmune blistering disease. These alternatives were considered less likely because of the unilateral prodromal neuropathic pain, progression from an initially localized eruption to widespread extra-dermatomal lesions, and positive VZV PCR from vesicle fluid and blood.

Intravenous acyclovir was initiated promptly at day of admission at a dose of 500 mg every 8 h, equivalent to approximately 8.1 mg/kg/dose based on body weight of 62 kg, with each dose infused over 60 min. This 500 mg fixed dose was selected as the initial regimen following pharmacy dose rounding. Gabapentin 300 mg orally three times daily was prescribed for neuropathic pain, and methylcobalamin 0.5 mg intramuscularly once daily was administered as adjunctive therapy. Topical treatment included compound polymyxin B ointment for pustular lesions and acyclovir cream for non-pustular lesions. Adequate hydration was maintained during intravenous acyclovir treatment, and no nephrotoxicity, neurotoxicity, or other treatment-related adverse effects were observed.

Within 48 h of antiviral treatment, no new lesions developed and systemic symptoms improved. After 2 days of intravenous therapy, acyclovir was transitioned to oral acyclovir 800 mg every 4 h while awake, five times daily, for an additional 7 days, resulting in a total antiviral-treatment duration of 9 days. By hospital day 4, the lesions had crusted, and pain intensity decreased from 9/10 to 3/10. She remained afebrile and stable during the rest of her hospitalization. The patient was discharged after 9 days in stable condition. At follow-up 4 weeks later, cutaneous lesions had resolved completely, neuropathic pain had markedly improved, and there was no evidence of postherpetic neuralgia or recurrent zoster. The patient reported that the initial pain was more distressing than the skin lesions and that the delayed diagnosis caused significant anxiety. She noted substantial relief after the correct diagnosis was made and antiviral treatment was started, and she agreed to publication of the case to help clinicians recognize similar presentations earlier.

## Discussion

HZ results from reactivation of the virus, which remains latent in dorsal root ganglia after primary infection. Reactivation typically results in unilateral, excruciating dermatomal vesicular eruptions. The condition is known as DHZ when vesicular lesions spread beyond the primary dermatome to include more than 20 lesions or multiple noncontiguous areas. Usually, HZ is limited to a single dermatome. Significant immunosuppression, such as that observed in patients with hematologic malignancies, HIV infection, organ transplantation, or immunosuppressive therapy, is usually linked to this severe form (1).

DHZ in immunocompetent individuals is extremely rare (6). Its diagnosis may be delayed or missed, especially when initial symptoms mimic other conditions, as in this case where the patient's dermatomal pain led to a misdiagnosis of lumbar disc herniation. Based on our literature review, several published reports have described the misdiagnosis of HZ with sciatica or radiculopathies in patients (2, 7–13), raising the importance of considering HZ, particularly in older patients (Table 2). Due to a delay in skin rash appearance, HZ may be misdiagnosed with several other conditions, including but not limited to acute appendicitis (14), orbital complication of acute sinusitis (4), biliary colic (15), cubital tunnel syndrome (16), acute abdomen (3), odontalgia (17), testicular torsion (18) as described previously in various case reports, illustrating the broad spectrum of clinical misdiagnosis that may occur before rash onset.

**Table 2:** Previously reported herpes zoster cases initially misdiagnosed as sciatica or radiculopathy.

References	Age (year), gender	Pain characteristics	Physical examination	Time from pain to rash	MRI findings	Misdiagnosis	Location of rash	Treatment	Recovery after HZ treatment
Koda et al. (2)	74, male	Left-sided sciatic pain upon standing or walking for 5 min, VAS score: 85/100	Sciatic pain provocation by extension and left bending of the trunk, no apparent motor weakness, sensory disturbance, or vesico-rectal disturbance, palpable bilateral dorsalis pedis artery	Approximately 1 month	MRI: spinal canal stenosis at L4-L5 level and redundant nerve roots superior to the most stenotic level	Sciatica induced by spinal canal stenosis	Dorsal and plantar surfaces of the great toe and lateral malleolus	NSAIDS + caudal epidural block (lidocaine) + steroid, then planned for decompressive surgery, famciclovir + NSAID after HZ diagnosis	1 week
Montgomery et al. (12)	67, female	Left upper extremity pain, weakness, numbness, and tingling and several recent falls, changes in balance, and decreased fine motor coordination and dexterity, no other abnormal upper motor neuron signs in the right upper extremity or bilateral lower extremities	Decreased strength (4/5) in the left wrist flexors, biceps, intrinsics, and triceps, diminished sensitivity to light touch in the radial aspect of the left forearm, thumb, index, and long finger, unilateral left-sided hyperreflexia graded as 3 at biceps, triceps, and brachioradialis, positive Hoffman sign of the left upper extremity	3 weeks	C5-C6 and C6-C7 spondylosis with corresponding central and neuro foraminal cervical stenosis	Cervical spondylotic myeloradiculopathy	an erythematous vesicular rash extending from the left shoulder to the first and second digits	valacyclovir and topical steroids	1-year postherpetic neuralgia
Hackenberg et al. (9)	62, male	Severe lumbar spine pain with radiation alongside the dermatome L5 on the right side to the first toe, no motoric deficit in both legs, no bladder or bowel dysfunction, and no saddle anesthesia	hypoesthesia in the same dermatome, positive femoral nerve stretch test and straight leg raise test (at 20 ° elevation) of the right leg, similar Achilles tendon reflex, and the patella tendon reflex on both sides	6 days	no herniated disc but aslight spinal stenosis between L4 and L5, and an unspecific swelling of the spinal nerve root L5 on the right side	Lower back pain with sciatic disorder	Lower back	Acyclovir + ibuprofen	1 week
Mallepally et al. (11)	30, male	Severe back pain, buttock pain radiating to the right anterior thigh associated with dysesthesia over the affected dermatome., VAS score: 8/10	Normal	7 days	MRI: a small disc bulge at L3-L4 level	Sciatica	Right anteromedial thigh and right paraspinal region near the posterior superior iliac spine	Acyclovir + pregabalin	3 weeks
Mallepally et al. (11)	45, female	Back pain and right leg pain with associated dysesthesia over the right anterolateral thigh and leg	NM	2 days	MRI: disc bulge at L4-L5 compressing the nerve root to a mild extent	Sciatica	Anterolateral thigh	Acyclovir + pregabalin	3 weeks
Wei et al. (13)	55, male	Radiation pain in the right lower limb -from the back of the right hip, behind the thigh, along the lateral crural region, and to the dorsum of the foot, VAS score: 8–9/10, no lower back pain, weak walking, no intermittent claudication	No abnormalities in the appearance of the spine and limb, positive L4-L5, L5-S1 spinous process gap tenderness, normal limb sensation, muscle strength, and muscle tension, knee reflex: left/right = + +/–; ankle reflex: left/right = ++/+; Lasegue sign: left/right = –/+(60?); Bragard sign: left/right = –/–; Femoral nerve tension test: left/right = -/-; Piriformis strain test: left/right = –/–	10 days	MRI: lateral type disc protrusion on the right side in L4-L5 (Modic II type degeneration), mildly pressing the lumbar 5^th^ nerve on the right	Sciatica caused by lumbar disc herniation	Right hip and right calf w	Famciclovir	3 months
Jeong et al. (10)	58, male	Constant sharp right-sided neck pain and tingling sensation with radiation to the right lateral arm, forearm, thumb, and index finger, AS score: 7/10	Decreased hand grasping power (manual motor testing: 3/5).	11 days	X-ray: degenerative change with reduced disc height at the C5–6 level MRI: foraminal stenosis with disc protrusion between the 5th and 6th cervical spine (C5/6) level and a high signal change at the same level of the spinal cord	Radiculopathy induced by cervical disc herniation with cord injury	Right arm (C6 dermatome)	Surgery, then Antiviral treatment + nerve block after HZ diagnosis	6 months
Dharmshaktu et al. (7)	52, male	Acute radiculopathy to his right thigh and upper leg, along with pain over the buttocks and lower back	NM	5 days	X-ray: no significant abnormality, acute disc prolapse case	Radiculopathy	Right upper gluteal area	Acyclovir	1 week
Essayh et al. (8)	60, male	Long-standing lower back pain and right buttock area with sudden onset irradiating pain to the right lower limb lingering behind the thigh, the outer side of the knee and the leg, the top of the foot arriving at the fourth and the fifth toes, no bowel or bladder symptoms	No loss of weakness of lower limbs	7 days	MRI: mild disc herniation between the 4th and 5th lumbar vertebrae with small root compression	Lumbar disc herniation	Right buttock area, the outer side of the knee and the leg, lateral malleolus, the top of the foot, the fourth and the fifth toes	Acyclovir	A few weeks

Intravenous acyclovir at 10 mg/kg every 8 h, administered over 1 h, is a commonly referenced regimen for severe or complicated VZV infection. In the present case, the initial pharmacy-rounded fixed dose of 500 mg every 8 h corresponded to approximately 8.1 mg/kg/dose and was therefore lower than the conventional 10-mg/kg weight-based dose. After 48 h of intravenous therapy, no new lesions were observed and the patient showed clear clinical improvement; treatment was therefore transitioned to oral acyclovir 800 mg every 4 h while awake, five times daily, for an additional 7 days. The total antiviral-treatment duration was 9 days. The oral regimen was consistent with the conventional dosing schedule for acute herpes zoster, although the initial intravenous dose was lower than the commonly recommended weight-based regimen.

It should be noted that DHZ is classically associated with impaired cell-mediated immunity, but it can rarely occur without overt immunosuppression. When the clinical presentation is atypical or disseminated, laboratory confirmation can strengthen the diagnosis. Once vesicular lesions are present, PCR testing of vesicle fluid or other lesion material is the preferred confirmatory approach because it directly samples the affected site. Blood PCR may support viremia, particularly in disseminated disease, but its diagnostic yield varies according to the blood fraction tested, timing of collection, host immune status, disease extent, and assay characteristics.

During the pre-eruptive phase, however, the diagnostic performance of VZV PCR is less certain and varies according to the specimen type, timing of sampling, assay characteristics, and host immune status. In patients with suspected herpes zoster, saliva PCR has shown greater sensitivity than plasma PCR, whereas lesion-based PCR has the highest diagnostic performance once cutaneous lesions are available. In a prospective study of patients with suspected herpes zoster, salivary VZV PCR had a sensitivity of 88% and plasma PCR a sensitivity of 28%, while the specificity of both tests was 100% (19). VZV DNA has also been detected in blood during the week preceding rash onset in 10 of 13 patients in a small retrospective series; however, 12 of these patients were immunosuppressed, limiting the generalizability of these findings to immunocompetent adults (20). In another prospective study involving immunocompetent patients with typical or atypical herpes zoster, VZV DNA was detected in all available blister-fluid samples, but only in 40% of saliva samples from typical cases and 20% from atypical cases, while all plasma samples were negative (21). Evidence regarding PCR before rash onset remains limited, particularly in immunocompetent individuals. Although VZV DNA has been detected in blood during the week preceding rash onset in some immunocompromised patients, these findings may not be generalizable to otherwise immunocompetent adults. Consequently, a negative VZV PCR result from blood, plasma, or saliva during the prodromal phase does not exclude herpes zoster and should not provide false reassurance. When clinical suspicion persists, close clinical reassessment is warranted, with lesion-based PCR performed if compatible cutaneous lesions subsequently emerge. In the present patient, PCR testing was performed after vesicular lesions had appeared; therefore, the limitations of pre-eruptive non-lesional PCR do not affect the strength of the lesion-based etiologic confirmation.

Table 2 summarizes previously reported cases in which herpes zoster initially mimicked sciatica or radiculopathy. Across these reports, pain typically preceded rash by several days to weeks, neuroimaging often showed incidental or mild degenerative abnormalities, and initial management frequently targeted presumed mechanical radiculopathy. These patterns parallel the present case and underscore the need to consider VZV reactivation when acute dermatomal pain is disproportionate to objective neurological findings.

This case adds to the limited literature on disseminated herpes zoster in apparently immunocompetent adults and highlights an uncommon diagnostic pathway in which prodromal neuropathic pain was initially attributed to lumbar disc disease. Published reports of herpes zoster initially misdiagnosed as radiculopathy have predominantly involved localized disease (2, 7–13); disseminated disease in an apparently immunocompetent adult appears to be exceptionally uncommon. By demonstrating that even disseminated forms of herpes zoster can manifest subtly, with initial symptoms limited to a single dermatome and easily confused with a structural spinal pathology, this case adds a distinctive dimension to the diagnostic spectrum of HZ reactivation. This presentation is uncommon in immunocompetent people, which emphasizes the need for clinical awareness and vigilance. Age is a well-established risk factor for HZ reactivation due to immunosenescence, a gradual decline in cell-mediated immunity with age. Our patient, although otherwise healthy, was 59 years old, suggesting a possible role for age-related immune decline even in the absence of clinically overt immunodeficiency. Essayh et al. and Mallepally et al. reported similar cases of HZ (but not in the disseminated form) in immunocompetent patients, proposing immune dysregulation as a contributing factor (8, 11).

Importantly, T-cell-mediated immunity is the primary defense against HZ reactivation. Several studies have shown that even in the absence of quantitative T-cell abnormalities, there may be qualitative impairments in cellular immune response. Previous studies found that disseminated zoster patients exhibited a significantly reduced CD4/CD8 ratio compared to both healthy controls and patients with localized herpes zoster, implying a degree of subclinical immune dysfunction (22, 23).

Although our patient's CD4/CD8 ratio and immunoglobulin levels were within normal ranges, it is impossible to completely rule out the possibility of temporary immune dysregulation.

An additional teaching point is that incidental degenerative spine findings may anchor clinical reasoning toward lumbar disc disease when the pain pattern is neuropathic and dermatomal. Pain intensified during the same period in which lumbar traction was administered; however, this temporal association does not establish causality, and the worsening pain may instead have reflected the natural progression of the herpes zoster prodrome.

A particularly notable aspect of this case was the atypical character of the prodromal pain. Instead of continuous or sustained dermatomal discomfort, the patient experienced brief, recurrent burning and needle-like paroxysms that shifted between the posterior thigh and calf. This unusual temporal and spatial pattern, together with the absence of rash and the presence of multilevel degenerative findings on lumbar CT, reduced the specificity of the early clinical presentation and may have contributed to the initial diagnosis of lumbar disc disease. Pain commonly precedes the cutaneous eruption in herpes zoster and may lead to misdiagnosis during the early phase (24). Even in the absence of rash, the dermatome-specific pattern of pain is a crucial indicator that, if identified, may lead to an earlier evaluation of HZ. This case highlights the necessity for clinicians to maintain a high index of suspicion for VZV reactivation in older adults presenting with unexplained dermatomal pain, particularly when there are no imaging findings to fully explain the symptoms. The severity and side effects of herpes zoster, such as postherpetic neuralgia and dissemination, can be considerably decreased with early antiviral treatment.

Methylcobalamin was administered in this case as an empirical adjunct according to local clinical practice rather than as a component of standard therapy for disseminated herpes zoster. Although small single-center randomized trials have reported analgesic benefits with local methylcobalamin-based injections in acute or subacute herpetic neuralgia, those studies used different routes and treatment protocols and do not establish routine intramuscular methylcobalamin as standard treatment for acute or disseminated herpes zoster. Therefore, no independent therapeutic effect can be attributed to methylcobalamin in the present case (25, 26).

DHZ is classically associated with impaired cell-mediated immunity, but this case shows that it may also occur in an older adult without an identified immunocompromising condition on routine evaluation. The case is particularly instructive because the initial presentation resembled lumbar radiculopathy, and degenerative lumbar imaging findings contributed to diagnostic anchoring.

Clinicians should consider herpes zoster in older adults presenting with acute unilateral burning, stabbing, or electric shock-like limb pain, particularly when neurological deficits are absent or minimal and imaging abnormalities do not fully explain symptom severity. Before cutaneous lesions appear, diagnosis relies primarily on clinical suspicion, careful examination, and close reassessment because lesion-based testing is not yet feasible. Once compatible skin lesions emerge, PCR testing of lesion material can provide etiologic confirmation. Separately, when herpes zoster is clinically suspected or confirmed, timely antiviral therapy may limit ongoing disease activity and reduce the risk or severity of complications; however, treatment does not reverse any diagnostic delay that has already occurred.

## Data Availability

The raw data supporting the conclusions of this article will be made available by the authors, without undue reservation.
